# Bacterial Isolates and Their Antimicrobial Susceptibility Patterns of Wound Infections among Inpatients and Outpatients Attending the University of Gondar Referral Hospital, Northwest Ethiopia

**DOI:** 10.1155/2017/8953829

**Published:** 2017-03-12

**Authors:** Aynalem Mohammed, Mengistu Endris Seid, Teklay Gebrecherkos, Moges Tiruneh, Feleke Moges

**Affiliations:** ^1^Department of Public and Clinical Microbiology, Amhara Public Health Institution, Dessie Branch, P.O. Box 286, Dessie, Ethiopia; ^2^School of Biomedical and Laboratory Sciences, College of Medicine and Health Sciences, University of Gondar, P.O. Box 196, Gondar, Ethiopia; ^3^Australian Institute of Tropical Health and Medicine, James Cook University, Cairns, QLD 4878, Australia

## Abstract

*Background.* The widespread uses of antibiotics, together with the length of time over which they have been available, have led to the emergence of resistant bacterial pathogens contributing to morbidity and mortality. This study was aimed to assess bacterial isolates and their drug susceptibility patterns from inpatients and outpatients with pus and/or wound discharge.* Methods.* A cross-sectional study was conducted at the University of Gondar Referral Hospital from March to May, 2014. Wound swab samples were collected from each study participant and inoculated into appropriate media. The bacterial pathogens were identified using standard microbiological methods. Antimicrobial susceptibility tests were performed using disk diffusion technique following Kirby-Bauer method.* Results.* A total of 137 study subjects were included in the study with bacterial isolation rate of 115 (83.9%). Of all, 81 (59.1%) were males. Seventy-seven (57%) of the isolates were Gram-negative and 59 (43%) were Gram-positive. From the total isolates,* Staphylococcus aureus* was the most predominant isolate 39/115 (34%) followed by Klebsiella species (13%), coagulase negative staphylococci spp. (12%) and* Pseudomonas aeruginosa*. Gram-positive isolates were resistant to ampicillin (86.4%), amoxicillin (83%), penicillin (81.3%), oxacillin (74.6%), and tetracycline (59.4%), while Gram-negative isolates were resistant to amoxicillin (97.4%), ampicillin (94.8%), tetracycline (72.7%), trimethoprim/sulfamethoxazole (66%), and chloramphenicol (54.5%).* Conclusion*. High prevalence of bacterial isolates was found,* Staphylococcus aureus* being the most dominant. High rates of multiple drug resistance pathogens to the commonly used antimicrobial agents were isolated. Therefore, concerned bodies should properly monitor the choice of antibiotics to be used as prophylaxis and empiric treatment in the study area.

## 1. Introduction

Wound provides a moist, warm, nutritive environment conducive to microbial colonization, proliferation, and infection [1–4]. Many different bacterial species live on human skin, in the nasopharynx, gastrointestinal tract, and other parts of the body with little potential for causing disease, because of the first line of defense within the body [5, 6]. Despite this, any breach in the skin surface whether trauma, accident, surgical operation, or burn provides an open door for bacterial infections [5].

The most common underlying event for all wounds is trauma [7]. Trauma may be accidental or intentionally induced. The category of intentionally induced trauma includes hospital-acquired wounds, which can be grouped according to how they are acquired, such as surgically and by using intravenous medical devices. The non-intentionally induced, hospital-acquired wounds can be the pressure sores [7]. The development of wound infection depends on the integrity and protective function of the skin, the number and types of organism and their synergy, the pathogenicity and virulence of the bacterial species, nature of surgery, use of antibiotics, and the immunocompetency of the host [8–11]. Infected wounds are characterized by bacterial burden, chronic inflammation, and an unbalanced cellular defense mechanism [12]. The common wound pathogens include bacteria, fungi, protozoa, and viruses [13]. Common bacterial pathogens associated with wound infection include* Staphylococcus aureus*,* Escherichia coli, Pseudomonas aeruginosa*,* Klebsiella pneumoniae*,* Streptococcus pyogenes*,* Proteus* species,* Streptococcus *species, and* Enterococcus *species [12, 14, 15].

Every year, millions of people experience burns, suffer from nonhealing wounds, or have acute wounds that become complicated by infections, dehiscence, or problematic scarring. Effective wound treatment requires carefully considered interventions often requiring multiple clinic or hospital visits and the resulting costs of wound care are staggering [16–18].

In developing countries, like Ethiopia, wound infections are major health problems [19]; large number of people die daily of preventable and curable wound infections [15]. These are serious problems in hospitals specially in surgical practices, where clean operations can become contaminated with virulent organisms and subsequently infected [6]. It is also a common practice that antibiotics can be purchased without prescription; this leads to misuse of antibiotics by the public, thus, contributing to the emergence and spread of antimicrobial resistance [20].

Advances in infection control have not completely eradicated these problems, because of the high prevalence of drug resistance pathogens [5]. The widespread and prolonged use of antibiotics lead to the emergence of resistant bacterial pathogens in wound infections contributing to high morbidity and mortality rates [21]. The antibiotics resistant pathogens are acquired from either health care setting environment, health care personnel, or inpatients [20]. Hospital-acquired infections are further complicated by increasing prevalence of multidrug resistant bacterial pathogens like methicillin-resistant* S. aureus* (MRSA), methicillin-resistant coagulase negative* Staphylococcus *(CoNS), vancomycin-resistant* Enterococcus *(VRE), and polymicrobial flora and fungi [14].

Wound infection is a major concern among healthcare practitioners, not only in terms of increased trauma to the patient but also in view of its burden on financial resources and the increasing requirement for cost-effective management within the healthcare system [22–24]. Knowledge of the causative agents of wound infection has proved to be helpful in the selection of empirical therapy, on infection control measures in health institution, and in formulating rationales of antibiotic policy [2, 10].

It is therefore important to identify antimicrobial resistant pathogens from both inpatient and outpatient isolates. Information on bacterial pathogens from inpatient and outpatient is limited in Ethiopia. Thus, the aim of this research was to determine the bacterial etiologies and antimicrobial susceptibility patterns and to compare the antimicrobial susceptibility patterns of in- and outpatients of wound swab culture attending the University of Gondar Referral Hospital.

## 2. Materials and Methods

### 2.1. Study Area, Design, and Period

A cross-sectional study was conducted from March to May, 2014, at the University of Gondar Referral Hospital, located in Gondar town in Amhara regional state, in Northwest Ethiopia, 742 km from Addis Ababa. The hospital is a tertiary level teaching and referral hospital catering more than 450 beds for inpatients and rendering referral health services for over 5 million inhabitants in Northwest Ethiopia. This large number of people from the surrounding zones and nearby regions visit the hospital for different medical services. The hospital consists of an operating room, intensive care unit (ICU), fistula center, and 13 different wards and outpatient departments. Unfortunately the hospital has no dedicated burn unit for caring of burn patients and as a result these patients are still managed in general surgical wards.

### 2.2. Study Population, Sample Size, Sampling Technique, and Data Collection

All patients with wound infections, who visited the University of Gondar Referral Hospital during the study period, were included in the study. A total of 137 study participants (38 from outpatient population and 99 from inpatient population) who developed wound infections during the study period were consecutively enrolled through convenient sampling technique. Sociodemographic characteristics like age, sex, educational background, occupation, residence, and patient setting were gathered using pretested structured questioner.

### 2.3. Sample Collection, Processing, and Culture Method

Wound was cleaned with normal saline and swab of wound secretion/pus, purulent exudates, or wound discharge was aseptically obtained using sterile cotton swab from each study participant. Specimen was collected on moistened cotton swab without contaminating with skin commensals and the swab was immersed in a container of Brain Heart Infusion (BHI) transport medium [25]. Soon after collection, each sample was transported to the bacteriology laboratory in the biomedical complex at the School of Biomedical and Laboratory Sciences.

The collected swab samples were inoculated onto MacConkey agar (Oxoid Ltd., Basingstoke, Hampshire, UK) and mannitol salt agar and pseudomonas agar media (cetrimide) and incubated at 37°C for 18–24 hours; blood agar plate (BAP) (Oxoid, Ltd.) and chocolate agar plate (CAP) were incubated in a humid, 5% CO_2_ atmosphere for 18–22 hours at 35°C–37°C. All the plates were incubated aerobically and initially examined for growth after 24 hrs, and the ones without growth were further incubated for up to 48 hrs.

### 2.4. Isolation, Identification, and Drug Susceptibility Testing of Bacteria

After obtaining pure colonies, further identifications were done by using the standard microbiological technique, which includes Gram stain, colony morphology, and biochemical tests (Oxoid, Ltd.). Species identification of the isolates was performed from pure colonies using classical biochemical tests according to the standard guidelines [26].

Antimicrobial susceptibility testing was carried out on each identified organism by disc diffusion method on Muller Hinton agar (MHA) and blood agar as recommended by the Clinical and Laboratory Standards Institute (CLSI) [27]. The isolates were tested against vancomycin (30 *μ*g), oxacillin (5 *μ*g), gentamicin (10 *μ*g), erythromycin (15 *μ*g), ciprofloxacin (5 *μ*g), ceftriaxone (30 *μ*g), trimethoprim-sulfamethoxazole (25 *μ*g), chloramphenicol (30 *μ*g), tetracycline (30 *μ*g), amoxicillin, ampicillin (10 *μ*g), penicillin (10 IU), and cloxacillin (5 *μ*g) [27]. The zones of inhibition were measured and compared with the guidelines [27].

#### 2.4.1. Multidrug Resistance (MDR)

MDR is defined as nonsusceptibility to at least one agent in three or more antimicrobial classes [28].

### 2.5. Quality Control

Data quality was maintained using a questionnaire translated from English to Amharic. Pretesting of the questionnaire was done for completeness and appropriateness before data collection. The reliability of the findings was guaranteed by implementing quality control measures throughout the whole processes of the laboratory work. The reference strains used as control were* E. coli* (ATCC 25922),* P. aeruginosa *(ATCC 27853), and* Staphylococcus aureus* (ATCC 25923) [26].

### 2.6. Data Analysis

Data were entered and analyzed using SPSS version 20. Simple frequency was applied to see the distribution of sociodemographic variables. Proportions of categorical variables were compared by Chi square. *P* value ≤ 0.05 was considered statistically significant.

### 2.7. Ethical Consideration

Ethical clearance was obtained from the ethical committee of School of Biomedical and Laboratory Sciences, College of Medicine and Health Sciences, University of Gondar. Official permission and written informed consent were obtained from University of Gondar Referral Hospital administration office and from each study participant, respectively. The assent of children (<18 years old) was obtained from their family or guardian. All the information obtained from each study participant was kept confidential. The laboratory result from the study participant was communicated to their doctors for appropriate treatment.

## 3. Results

A total of 137 study participants with wound infection were included in the study. Among these, 81 (59.1%) were males and 56 (40.9%) were females with the age range of 2 to 80 years and mean age of 31.63 ± 15.39 years. The majority of the study participants (67.9%) were in the age groups of 16–40 years, and 86 (62.8%) lived in rural areas, of whom 65 (47.4%) were unable to read and write (Table 1).

### 3.1. Magnitude of Bacterial Etiologic Agents of Wound Infection

Of the 137 study participants (99 inpatients and 38 outpatients) with wound infection, 115 (83.9%) of them showed bacterial growth. Gram-negative bacterial spp. were commonly isolated, 77 (56.6%), versus the Gram-positive bacterial spp., 59 (43.4%). A total of 136 bacterial pathogens were recovered. Twenty-one of the swab cultures (18.3%) showed mixed growth, while the 94 (81.7%) showed single bacterial growth. The remaining 22 (16.1%) had no bacterial growth. The most prevalent wound type was postoperative (28%) followed by trauma (25%) and the least was Osteomyelitis (0.9%).* S. aureus *were the most frequently isolated bacteria accounting for 39 (28.7%) followed by* Klebsiella* spp. (17; 12.5%), CoNS (16; 11.8%),* Citrobacter* spp. (5; 11%),* Enterobacter* spp. (13; 9.6%),* P. aeruginosa* and* E. coli *(each 8; 5.9%), and* Proteus* spp. (6; 4.4%) (Table 2).

### 3.2. Antimicrobial Susceptibility Patterns of Bacterial Isolates from Wound Culture

The antimicrobial susceptibility patterns of the Gram-positive and Gram-negative bacterial isolates were presented in Tables 3 and 4, respectively. The predominant isolate,* S. aureus,* revealed high level of resistance to amoxicillin 34 (87.2%), penicillin 33 (84.6%), oxacillin 30 (76.9%), tetracycline 25 (64.1%), and erythromycin 24 (61.5%); and it was also found to be sensitive to gentamicin 32 (82.1%), ciprofloxacin and ceftriaxone, each 31 (81.2%), chloramphenicol 30 (76.9%), cloxacillin 27 (69.2%), and trimethoprim-sulfamethoxazole 24 (61.5%). All of the isolates of* S. aureus *were sensitive to vancomycin 39 (100%). One isolate of* Enterococcus *spp. was found to be resistant to vancomycin (VRE).

The second predominant Gram-negative isolate,* Klebsiella* spp., showed also high level of resistance to ampicillin 16 (94.1%), chloramphenicol 12 (70.6%), trimethoprim-sulfamethoxazole 11 (64.7%), ciprofloxacin 12 (58.8%), and ceftriaxone 9 (52.9%), but it was only sensitive to gentamicin 12 (70.6%).* E*.* coli* were resistant to ampicillin 6 (75%) and tetracycline 5 (62.5%), whereas most isolates of* E. coli* were sensitive to gentamicin 7 (87.5%), ceftriaxone 7 (87.5%), chloramphenicol 6 (75%), and ciprofloxacin 5 (62.5%).

*Pseudomonas aeruginosa* isolates were sensitive to gentamycin (62.5%) and ciprofloxacin. All isolates of* Proteus* spp. were resistant to tetracycline and ampicillin, each 6 (100%), trimethoprim-sulfamethoxazole 5 (83.3%), and chloramphenicol 3 (50%), whereas 5 (83.3%) of isolates were sensitive to ceftriaxone. All isolates of* Salmonella arizonae* were 4 (100%) resistant to tetracycline, ampicillin, gentamicin, and trimethoprim-sulfamethoxazole and this was also less resistant to chloramphenicol 2 (50%) and ceftriaxone 3 (75%). However, All isolates were sensitive to ciprofloxacin 4 (100%) (Table 4).

### 3.3. Comparison of Antimicrobial Resistance Patterns of Isolates from Inpatients and Outpatients

The most isolated* S. aureus *exhibiting resistance to penicillin, amoxicillin, oxacillin, erythromycin, tetracycline, trimethoprim-sulfamethoxazole, cloxacillin, chloramphenicol, gentamicin, and ciprofloxacin were 91.3%, 91.3%, 87%, 82.6%, 73.9%, 65.2%, 43.4%, 39.9%, 30.4%, and 34.8% isolates from inpatients and 68.8%, 81.2%, 62.5%, 31.2%, 50%, 0%, 12.5%, 0%, 0%, and 0% isolates from outpatients, respectively.

Coagulase negative* Staphylococcus *(CoNS) isolated from inpatient showed more resistance to amoxicillin (90.9%), penicillin (83.3%), oxacillin (75%), tetracycline (72.7%), trimethoprim-sulfamethoxazole, cloxacillin and erythromycin (63.6%), and chloramphenicol (54.5%) than from outpatient (Table 5).* E. coli* isolated from inpatients was resistant to tetracycline (100%) and trimethoprim-sulfamethoxazole and ampicillin (66.7%) each whereas outpatient isolates were resistant to tetracycline and chloramphenicol (40%) each, ampicillin (80%), and ciprofloxacin and trimethoprim-sulfamethoxazole each (40%). Chloramphenicol was (100%) sensitive to inpatient isolates whereas outpatient isolates are only sensitive to 60% of the strains (Table 5).

Gram-positive isolate from inpatient showed more resistance than that of outpatients for many drugs. However, vancomycin showed no difference in being inpatient and being outpatient (Table 6). Antimicrobial agents such as tetracycline, ceftriaxone, ampicillin, gentamicin, and trimethoprim-sulfamethoxazole were significantly associated (*P* < 0.05) with being in- and outpatient for Gram-negative isolates (Table 7). On the other hand, antimicrobial agents such as chloramphenicol and ciprofloxacin were not significantly associated (*P* > 0.05) for Gram-negative isolates.

The overall prevalence of multidrug resistance (MDR) patterns was 130 (95.5%). The results of multiple antimicrobial resistant patterns were presented in Table 8. Of the 39 (28.7%) isolates,* S. aureus *36 (94.8%) showed multidrug resistance to three or more antibiotics, while only one isolate of them was sensitive to all antimicrobial agents tested. The thirteen isolates of CoNS were found to be resistant to more than five antibiotics. Among two isolates of* S. pyogenes* only one was found to be resistant to one antibiotic. The two isolates of* Enterococcus *spp. were found to be resistant to more than five antibiotics. Of the Gram-negative bacteria* Salmonella arizonae* showed high level of MDR to more than five antimicrobial agents tested. Of the eight* E. coli *isolates five were resistant to more than five antimicrobials.

## 4. Discussion

Among 137 study subjects, bacterial pathogens were isolated from 115 patients with the isolation rate of 83.9%. This was higher than the previous study done in Gondar (52%), Bahir Dar (53%), Dessie (70.5%), and Addis Ababa (42%), Ethiopia [15, 19, 29, 30]. This high rate of bacterial isolation in the present study may be due to the differences of the quality of wound swab specimens and bacteriological techniques (overnight incubation in BHI) used. On the other hand the type of wound pathogens and their rate of isolation in these findings were found to be consistent with study conducted in India (79%) [31]. However, it was lower than a study done in Nigeria (94%) [17].

According to the present study, 94 (81.7%) of the wound swab cultures showed monomicrobial growth, while the remaining 21 (18.3%) revealed polymicrobial growth. This finding is consistent with a retrospective study done in Gondar [15]. The reason may be chronic wounds tending to show monomicrobial infections. The present study revealed polymicrobial infections, mainly by* Klebsiella *spp. and* S. aureus*, which is consistent with report from India [31].

The current findings showed that the rates of isolation of Gram-negative and Gram-positive were 56.6% and 43.4%, respectively. This was in agreement with studies done in Zaria, Nigeria, 55% and 44%, respectively [32]. However, the present result is different from the previous report from Gondar University Hospital, Ethiopia (29% versus 71%) [15]. The present findings show higher rates of isolation of Gram-negative wound pathogens from the same area. This high rate of Gram-negative and low rate of Gram-positive isolates from wound in the same area may be due to high number of cases included from inpatients in the present study compared to outpatients. This may probably contribute high number of Gram-negatives than Gram-positives.

The predominant isolate in the present study was found to be* S. aureus*, which was 34%; this finding was higher when compared with previous reports from Italy (28.2%) [7] and Nigeria (25.1% and 25%) [2, 5]. This difference may be due to improved facilities of the hospital management from these countries in the infection prevention and control program. However, it is lower than reports in Nigeria (44%) and other parts of Ethiopia Dessie (41.6%), Bahir Dar (69.7%), and Gondar (65.5%) [15, 19, 30, 32]. The second predominant Gram-negative bacterium in this study was* Klebsiella* spp. 17 (12.5%). Similar result was reported from Cape, South Africa, which revealed that* K. pneumonia* were the second predominant organisms isolated (13.4%) [33].

In this study CoNS accounted for 11.8% of the isolates. This finding is similar to a report in Nigeria, where* S. epidermidis *accounted for 11.4% [5]. The percentage of* Citrobacter* spp. 15 (11%) is higher than the previous studies in Gondar University Teaching Hospital 1 (1.3%), Bahr Dar 2 (0.9%), and Dessie Ethiopia 21 (4.2%) [15, 19, 30]. Among the 15 isolates of* Citrobacter* spp. 100% showed MDR and this alarms that multiple drug resistant strains of* Citrobacte*r spp. circulate in the study area. In the present study the susceptibility pattern of* S. aureus* isolates demonstrated high level of resistance to the commonly used antimicrobial agents. This result is in agreement with a study done in Jimma [34]. The present study showed a single isolate vancomycin-resistant* Enterococcus spp*. (VRE), indicating the emergence of VRE may pose therapeutic problems.

Oxacillin-resistant CoNS has become the predominant pathogen. According to the current study oxacillin-resistant CoNS were 12 (75%); this is in line with a study reported in Nigeria (77.3%) [21]. The percentage of isolates that were resistant to cloxacillin was 38.9% which was similar to a study done in Addis Ababa, Ethiopia (37.2%) [29]. Showing that, they may be reservoirs for methicillin-resistant* Staphylococcus aureus, *as they are common nosocomial wound infections. However, this finding was inconsistent with a report in Nigeria (98.3%) [5]. Ciprofloxacin was relatively sensitive for both Gram-positive and Gram-negative isolates except* Klebsiella* spp. However, level of resistance to ciprofloxacin is increasing from 16% in 2006 [15] to 36% in the present study in the same study area.

The present study demonstrated that amoxicillin was resistant to 83% of Gram-positives which was higher than a study done in Dessie reported as amoxicillin had the highest resistance rate 78.9% [30]. This sharp increase resistance patterns may be due to overuse of it as empiric treatment option for most of the patients. The current finding also showed that* S. aureus* isolated from inpatients was more resistant than from outpatients. Similarly, a study done in Jimma reported that inpatient isolates of* S. aureus *were more resistant than outpatient isolates to all the tested antibiotics except erythromycin [34].

Overall MDR patterns of the isolated pathogens were 130 (95.5%), this is in line with the studies conducted in Bahir Dar, Ethiopia, 95.5% [19] and higher than previous study in 2006 which was 78.5% [15]. This may be due to massive use of antimicrobials in the area without prescription and as empirical treatment option by physicians or prolonged use of antibiotics may be responsible for the development of more resistant strains of the pathogens.

## 5. Conclusions

The isolation rate of bacterial pathogens was high. The predominant isolates were* S. aureus, Klebsiella *spp., CoNS*, Citrobacter* spp.,* Enterobacter* spp.,* P. aeruginosa,* and* E. coli.* The present findings show higher rates of isolation of Gram-negative wound pathogens compared to Gram-positives. Most Gram-positive isolates were sensitive to vancomycin, gentamicin, and ciprofloxacin but resistant to penicillin, tetracycline, and oxacillin.

Alarmingly high rate of MDR to commonly used antibiotics from wound infection were reported. Continuous surveillance is necessary to guide appropriate therapy for wound infection and rational use of antimicrobial agents should be sought to prevent the emergence of MDR pathogens.

## Figures and Tables

**Table 1:** Sociodemographic characteristics of study participants with wound swab cultures among inpatients and outpatients attending the University of Gondar Referral Hospital from March to May, 2014.

Characteristics	Frequency	Percent
*Sex *		
Male	81	59.1
Female	56	40.9
*Age in ( years)*		
≤15	14	10.2
16–40	93	67.9
41–60	23	16.8
≥61	7	5.1
*Educational status*		
No formal education	65	47.4
Primary school (1–8)	47	34.3
Secondary school (9–12)	18	13.1
College/university	7	5.1
*Residence*		
Urban	51	37.2
Rural	86	62.8
*Occupation*		
Farmer	57	41.6
Housewife	24	17.5
Daily labor	10	7.3
Student	15	10.9
Others^*∗*^	31	22.6
*Patient setting*		
Inpatient	99	72.8
Outpatient	38	27.2

*Overall *	*137*	*100*

^*∗*^Jobless, beiger, driver, carpenter, merchant, and civil servant.

**Table 2:** Bacterial isolates of wound infection among inpatients and outpatients attending the University of Gondar Referral Hospital from March to May, 2014.

Bacterial isolates	Inpatient *n* (%)	Outpatient *n* (%)	Frequency *n* (%)
*S. aureus*	23 (23.7)	16 (41)	39 (28.7)
CoNS	11 (11.4)	5 (13)	17 (12.5)
*P. aeruginosa*	8 (8.3)	0 (0)	16 (11.8)
*E. coli*	3 (3)	5 (12.8)	15 (11)
*Proteus *spp.	4 (4.1)	2 (5.1)	13 (9.6)
*Klebsiella *spp.	13 (13.4)	4 (10.3)	8 (5.9)
*Salmonella arizonae*	4 (4.1)	0 (0)	8 (5.9)
*Serratia *spp.	3 (3)	0 (0)	6 (4.4)
*Enterobacter *spp.	10 (10.3)	3 (7.7)	4 (2.9)
*Citrobacter *spp.	13 (13.4)	2 (5.1)	3 (2.2)
*Enterococci *spp.	2 (2)	0 (0)	2 (1.5)
*S. pyogenes*	0 (0)	2 (5.1)	2 (1.5)
*Acinetobacter *spp.	1 (1.03)	0 (0)	2 (1.5)
*Achromobacter *spp.	2 (2.06)	0 (0)	1 (0.7)

*Total*	*97 (100)*	*39 (100)*	* 136 (100)*

CoNS = coagulase negative *Staphylococcus*. *n = *number.

**Table 3:** Antimicrobial susceptibility patterns of Gram-positive bacterial isolates from wound swab cultures among inpatients and outpatients attending the University of Gondar Referral Hospital from March to May, 2014.

Bacterial isolates	Number of resistance pathogens to antimicrobial agents (%)											
	VAN	OXA	CXC	PEN	E	TE	C	CRO	AML	CN	CIP	SXT
*S. aureus * (*n* = 39)	0 (0)	30 (76.9)	12 (30.8)	33 (84.6)	24 (61.5)	25 (64.1)	9 (23.1)	8 (20.5)	34 (87.2)	7 (17.9)	8 (20.6)	15 (38.5)
CoNS (*n* = 16)	0 (0)	12 (75)	9 (56.2)	13 (81.2)	8 (50)	10 (62.5)	6 (37.5)	8 (50)	13 (81.2)	3 (18.8)	3 (18.8)	7 (43.8)
*Enterococci *spp. (*n* = 2)	1 (50)	2 (100)	2 (100)	2 (100)	1 (50)	0 (0)	0 (0)	1 (50)	2 (100)	1 (50)	1 (50)	1 (50)
*S. pyogenes * (*n* = 2)	0 (0)	0 (0)	0 (0)	0 (0)	0 (0)	0 (0)	0 (0)	0 (0)	0 (0)	1 (50)	0 (0)	0 (0)

*Total (59)*	*1 (1.6)*	*44 (74.6)*	*23 (38.9)*	*48 (81.3)*	*33 (55.9)*	*35 (59.4)*	*15 (25.4)*	*17 (28.8)*	*49 (83)*	*12 (20.3)*	*12 (20.3)*	*23 (38.9)*

VAN: vancomycin, OXA: oxacillin, CXC: cloxacillin, PEN: penicillin, E: erythromycin, TE: tetracycline, C: chloramphenicol, CRO: ceftriaxone, AML: amoxicillin, CN: gentamicin, CIP: ciprofloxacin, SXT: trimethoprim-sulfamethoxazole, and CoNS: coagulase negative *Staphylococcus*. *n*: number of isolates.

**Table 4:** Antimicrobial susceptibility patterns of gram negative Bacteria isolated from wound swab cultures of patients among inpatients and outpatients attending the University of Gondar Referral hospital from March to May, 2014.

Bacterial isolates	Number of resistance pathogens to antimicrobial agents (%)						
	TE	C	CRO	AMP	CN	CIP	SXT
*Klebsiella* spp. (*n* = 17)	11 (64.7)	12 (70.6)	9 (52.9)	16 (94.1)	5 (29.4)	10 (58.8)	11 (64.7)
*Citrobacter *spp. (*n* = 15)	8 (53.3)	7 (46.7)	4 (26.7)	15 (100)	8 (53.3)	3 (40)	9 (60)
*Enterobacter *spp. (*n* = 13)	11 (84.6)	6 (46.2)	7 (53.8)	13 (100)	8 (61.5)	3 (23.1)	7 (53.8)
*E. coli * (*n* = 8)	5 (62.5)	2 (25)	1 (12.5)	6 (75)	1 (12.5)	3 (37.5)	4 (50)
*P. aeruginosa* (*n* = 8)	ND	6 (75)	3 (37.5)	ND	3 (37.5)	3 (37.5)	ND
*Proteus *spp. (*n* = 6)	6 (100)	3 (50)	1 (16.7)	6 (100)	2 (33.4)	1 (16.7)	5 (83.3)
*Salmonella arizonae* (*n* = 4)	4 (100)	2 (50)	3 (75)	4 (100)	4 (100)	0 (0)	4 (100)
*Serratia* spp. (*n* = 3)	2 (66.7)	1 (33.3)	3 (100)	3 (100)	2 (66.7)	2 (66.7)	3 (100)
*Achromobacter *spp. (*n* = 2)	0 (0)	2 (100)	2 (100)	2 (100)	2 (100)	2 (100)	0 (0)
*Acinetobacter *spp. (*n* = 1)	1 (100)	1 (100)	1 (100)	1 (100)	1 (100)	1 (100)	0 (0)

*Total *(*n* = 77)	*48 (62.3)*	*42 (54.5)*	*34 (44)*	*66 (85.7)*	*36 (46.7)*	*28 (36)*	*43 (55.8)*

TE: tetracycline, C: chloramphenicol, CRO: ceftriaxone, AMP: ampicillin, AML: amoxicillin, CN: gentamicin, CIP: ciprofloxacin, SXT: trimethoprim-sulfamethoxazole, *n*: number of isolates, and ND: not done.

**Table 5:** Antimicrobial resistance isolates of bacterial isolates from wound swab cultures among inpatients and outpatients attending the University of Gondar Referral Hospital from March to May, 2014.

Bacteria isolates	Patient settings	Percentage of number of resistance pathogens to antimicrobial agents *n* (%)												
		VAN	OXA	PEN	CXC	E	TE	C	CRO	AMP	AML	CN	CIP	SXT
*S. aureus*	IP (*n* = 23)	0 (0)	20 (87)	21 (91.3)	10 (43.4)	19 (82.6)	17 (73.9)	9 (39.1)	8 (34.8)	ND	21 (91.3)	7 (30.4)	8 (34.8)	15 (65.2)
	OP (*n* = 16)	0 (0)	10 (62.5)	11 (68.8)	2 (12.5)	5 (31.2)	8 (50)	0 (0)	0 (0)	ND	13 (81.2)	0 (0)	0 (0)	0 (0)
CoNS	IP (*n* = 11)	0	9 (75)	10 (83.3)	7 (63.6)	7 (63.6)	8 (72.7)	6 (54.5)	7 (63.6)	ND	10 (90.9)	3 (27.3)	3 (27.3)	7 (63.6)
	OP (*n* = 5)	0	3 (25)	3 (60)	2 (22.2)	1 (20)	2 (40)	1 (20)	2 (20)	ND	3 (60)	0 (0)	0 (0)	0 (0)
*E.coli*	IP (*n* = 3)	ND	ND	ND	ND	ND	3 (100)	0 (0)	1 (33.3)	2 (66.7)	ND	1 (33.3)	1 (33.3)	2 (66.7)
	OP (*n* = 5)	ND	ND	ND	ND	ND	2 (40)	2 (40)	0 (0)	4 (80)	ND	0 (0)	2 (40)	2 (40)
*Klebsiella* spp.	IP (*n* = 13)	ND	ND	ND	ND	ND	10 (76.9)	10 (76.9)	8 (61.5)	13 (100)	ND	5 (38.5)	9 (69.2)	11 (84.6)
	OP (*n* = 4)	ND	ND	ND	ND	ND	1 (25)	2 (50)	1 (25)	3 (75)	ND	0 (0)	1 (25)	0 (0)
Citrobacter spp.	IP (*n* = 13)	ND	ND	ND	ND	ND	8 (61.5)	6 (53.8)	4 (30.8)	13 (100)	ND	6 (60)	3 (30)	9 (69.2)
	OP (*n* = 2)						2 (0)	0 (0)	0 (0)	2 (100)		4 (40)	0 (0)	0 (0)
*Enterobacte*r spp.	IP (*n* = 10)	ND	ND	ND	ND	ND	8 (80)	5 (50)	8 (70)	12 (100)	ND	4 (40)	4 (40)	6 (85.7)
	OP (*n* = 3)						3 (100)	1 (33.3)	0 (0)	3 (100)		0 (0)	0 (0)	1 (33.3)
*Proteus *spp.	IP (*n* = 4)	ND	ND	ND	ND	ND	4 (100)	2 (50)	2 (40)	4 (100)	ND	1 (25)	1 (25)	3 (75)
	OP (*n* = 2)						2 (100)	1 (50)	0 (0)	2 (100)		1 (50)	0 (0)	2 (100)
Others	IP^*∗*^ (20)	1 (5)	2 (10)	2 (10)	2 (10)	1 (5)	15 (75)	12 (60)	13 (65)	19 (95)	ND	13 (65)	9 (45)	16 (80)
	OP^*∗∗*^ (2)	0 (0)	0 (0)	0 (0)	0 (0)	0 (0)	0 (0)	0 (0)	0 (0)	0 (0)		1 (50)	0 (0)	0 (0)

Total	**IP (97)**	**1 (1)**	**31 (31.9)**	**33 (34)**	**19 (19.6)**	**27 (27.8)**	**73 (75)**	**50 (51.5)**	**51 (53)**	**63 (64.9)**	**31 (31.9)**	**40 (41)**	**38 (39)**	**69 (71)**
	**OP (39)**	**0 (0)**	**13 (33.3)**	**14 (35.9)**	**4 (10.2)**	**6 (15.4)**	**20 (51)**	**7 (17.9)**	**3 (7.6)**	**14 (35.8)**	**26 (66.7)**	**6 (15.4)**	**3 (7.7)**	**5 (12.8)**

IP = inpatient; OP = outpatient ^*∗*^*P. aeruginosa, S. arizonae*, *Serratia *spp., *Achromobacter* spp., *Acinetobacter *spp., and *Enterococcus *spp. ^*∗∗*^*S. pyogene*s, VAN: vancomycin, OXA: oxacillin, CXC: cloxacillin, PEN: penicillin E: erythromycin, TE: tetracycline, C: chloramphenicol, CRO: ceftriaxone, AMP: ampicillin, AML: amoxicillin, CN: gentamicin, CIP: ciprofloxacin, SXT: trimethoprim-sulfamethoxazole, *n*: number of isolates, CoNS = coagulase negative *Staphylococcus*. and ND: not done.

**Table 6:** Comparison of antimicrobial resistant patterns of Gram-positive isolates among inpatients and outpatients attending the University of Gondar Referral Hospital from March to May, 2014.

Antimicrobials	Pattern	Bacterial isolates		*X*^2^	*P* value
		Inpatient (*n* = 36)	Outpatients (*n* = 23)		
Vancomycin	S	35 (97.2)	23 (100)	0.65	0.42
	R	1 (2.8)	0 (0)		
Oxacillin	S	5 (13.9)	10 (43.5)	6.48	0.01
	R	31 (86.1)	13 (56.5)		
Penicillin	S	2 (18.2)	9 (39.1)	10.4	0.001
	R	34 (70.8)	14 (60.8)		
Cloxacillin	S	17 (47.2)	19 (82.6)	7.3	0.007
	R	19 (52.8)	4 (17.4)		
Erythromycin	S	9 (25)	17 (73.9)	13.6	0.00
	R	27 (75)	6 (26)		
Tetracycline	S	11 (30.6)	13 (56.5)	3.9	0.048
	R	25 (69.4)	10 (43.5)		
Chloramphenicol	S	21 (48.8)	22 (51.2)	9.8	0.002
	R	15 (41.7)	1 (6.2)		
Ceftriaxone	S	21 (58.3)	22 (95.6)	9.8	0.002
	R	15 (41.6)	1 (4.3)		
Ampicillin	S	2 (5.6)	6 (26.1)	5.0	0.025
	R	34 (94.4)	17 (73.9)		
Amoxicillin	S	3 (8.3)	7 (30.4)	4.8	0.027
	R	33 (91.7)	16 (69.6)		
Gentamycin	S	25 (69.4)	22 (95.6)	5.9	0.015
	R	11 (30.6)	1 (4.4)		
Ciprofloxacin	S	24 (66.7)	23 (100)	9.6	0.002
	R	12 (33.3)	0 (0)		
Trimethoprim-sulfamethoxazole	S	13 (36.1)	23 (100)	24	0.00
	R	23 (63.9)	0 (0)		

S = sensitive, R = resistance, and *n* = number of isolates.

**Table 7:** Comparison of antimicrobial resistant patterns of Gram-negative isolates among inpatients and outpatients attending the University of Gondar Referral Hospital from March to May, 2014.

Antimicrobials	Pattern	Bacterial isolates		*X*^2^	*P* value
		Inpatient (*n* = 61)	Outpatients (*n* = 16)		
Tetracycline	S	13 (21.3)	8 (50)	5.2	0.02
	R	48 (78.7)	8 (50)		
Chloramphenicol	S	25 (41)	10 (62.5)	2.3	0.12
	R	36 (59)	6 (37.5)		
Ceftriaxone	S	28 (45.9)	15 (93.8)	11.7	0.000
	R	33 (54.1)	1 (6.2)		
Ampicillin	S	1 (1.6)	2 (12.5)	3.9	0.04
	R	60 (98.4)	14 (87.5)		
Amoxicillin	S	2 (3.3)	0 (0)	0.5	0.4
	R	59 (96.7)	16 (100)		
Gentamycin	S	28 (45.9)	13 (31.7)	6.3	0.012
	R	33 (54.1)	3 (18.7)		
Ciprofloxacin	S	36 (59)	13 (81.2)	2.7	0.1
	R	25 (41)	3 (18.8)		
Trimethoprim-sulfamethoxazole	S	15 (24.6)	11 (68.8)	11	0.001
	R	46 (75.4)	5 (31.2)		

S = sensitive, R = resistance, and *n* = number of isolates.

**Table 8:** Multidrug resistant patterns in bacterial pathogens isolated from wound swab cultures among inpatients and outpatients attending the University of Gondar Referral Hospital from March to May, 2014.

Antimicrobial classes related to number (%)						
Bacterial isolates	Number	*R*_1_	*R*_2_	*R*_3_	*R*_4_	≥*R*_5_
*S. aureus*	39 (28.7)	1 (2.6)	1 (2.6)	3 (7.7)	6 (15.4)	27 (69.2)
*Klebsiella* spp.	17 (12.5)	1 (5.9)	1 (5.9)	1 (5.9)	3 (17.6)	11 (64.7)
CoNS	16 (11.8)	1 (6.3)	2 (12.5)	0	0	13 (76.5)
*Citrobacter *spp.	15 (11)	0	2 (18.1)	4 (36.4)	2 (18.1)	7 (63.4)
*Enterobacter* spp.	13 (9.6)	0	2 (15.4)	1 (7.7)	1 (7.7)	9 (6.9)
*P. aeruginosa*	8 (5.9)	0	0	2 (25)	1 (12.5)	5 (62.5)
*E. coli*	8 (5.9)	0	3 (37.5)	0	0	5 (62.5)
*Proteus* spp.	6 (4.4)	0	0	0	1 (16.7)	5 (83.3)
*Salmonella arizonae*	4 (2.9)	0	0	0	0	4 (100)
*Serratia* spp.	3 (2.2)	0	0	0	1 (33.3)	2 (66.7)
*Enterococcus *spp.	2 (1.5)	0	0	0	0	2 (100)
*S. pyogenes*	2 (1.5)	0	0	0	0	0
*Achromobacter* spp.	2 (1.5)	0	0	0	0	2 (100)
*Acinetobacter* spp.	1 (0.73)	0	0	0	0	1 (100)

Total	136 (100)	4 (2.9)	11 (8)	9 (6.6)	14 (10.3)	93 (70.6)

*R*_1_–≥ *R*_5_ = resistance of bacteria to 1, 2, 3, 4, 5, and above classes of antimicrobials tested, and CoNS = coagulase negative *Staphylococcus*.

## Additional Points

*Limitation of the Study*. Due to resources limitation, we were not able to characterize each of the isolates at their species level like* Klebsiella* spp., CoNS,* Enterococcus *spp.,* Achromobacter* spp.,* Proteus* spp., and* Citrobacter *spp. We were also unable to perform Minimum inhibitory concentration (MIC) for vancomycin.
